# Association of MicroRNAs with Antibody Response to *Mycoplasma bovis* in Beef Cattle

**DOI:** 10.1371/journal.pone.0161651

**Published:** 2016-08-18

**Authors:** Eduardo Casas, Guohong Cai, Larry A. Kuehn, Karen B. Register, Tara G. McDaneld, John D. Neill

**Affiliations:** 1 USDA, ARS, National Animal Disease Center, Ames, IA 50010, United States of America; 2 USDA, ARS, U.S. Meat Animal Research Center, Clay Center, NE 68933, United States of America; University of Texas MD Anderson Cancer Center, UNITED STATES

## Abstract

The objective of this study was to identify microRNAs associated with a serum antibody response to *Mycoplasma bovis* in beef cattle. Serum from sixteen beef calves was collected at three points: in summer after calves were born, in fall at weaning, and in the following spring. All sera collected in the summer were ELISA-negative for anti-*M*. *bovis*. By the fall, eight animals were seropositive for IgG (positive group), while eight remained negative (negative group). By spring, all animals in both groups were seropositive. MicroRNAs were extracted from sera and sequenced on the Illumina HiSeq next-generation sequencer. A total of 1,374,697 sequences mapped to microRNAs in the bovine genome. Of these, 82% of the sequences corresponded to 27 microRNAs, each represented by a minimum of 10,000 sequences. There was a statistically significant interaction between ELISA response and season for bta-miR-24-3p (*P* = 0.0268). All sera collected at the initial summer had a similar number of copies of this microRNA (*P* = 0.773). In the fall, the positive group had an increased number of copies when compared to the negative group (*P* = 0.021), and this grew more significant by the following spring (*P* = 0.0001). There were 21 microRNAs associated (*P*< 0.05) with season. These microRNAs could be evaluated further as candidates to potentially improve productivity in cattle. The microRNAs bta-let-7b, bta-miR- 24-3p, bta-miR- 92a, and bta-miR-423-5p, were significatly associated with ELISA status (*P*< 0.05). These microRNAs have been recognized as playing a role in the host defense against bacteria in humans, mice, and dairy cattle. Further studies are needed to establish if these microRNAs could be used as diagnostic marker or indicator of exposure, or whether intervention strategies could be developed as an alternative to antibiotics for controlling disease due to *M*. *bovis*.

## Introduction

Bovine respiratory disease complex is the most expensive condition in cattle, costing up to $1 billion annually in the United States [1]. With infectious diseases being a major economical factor influencing productivity in the cattle industry, it is suggested that perhaps it is time to look for ways to reduce losses by focusing on the animal’s response to related pathogens, instead of continuing to focus on the pathogens themselves [2].

*Mycoplasma bovis* has been identified as an important pathogen causing respiratory disease of cattle [3, 4]. In cattle, the most common pathogen retrieved from lungs is is *M*. *bovis*, however, additional Mycoplasmas are retrieved, including *M*. *bovis*, *M*. *arginini*, *M*. *bovirhinis*, and *M*. *bovigenitalium* [5].

Cattle infected by *M*. *bovis* are usually chronically affected, unresponsive to treatment, and unable to attain commercial weights.

MicroRNAs have been proposed as a source of biomarkers and as indicators of exposure to pathogens [6, 7]. MicroRNAs are small non-coding RNAs that alter the transcriptome by inhibiting translation of messenger RNA, or by degrading them [8, 9]. MicroRNAs were first described in *C*. *elegans* in the 1990’s [10], and their origin and function in regulation of biological functions has been established [8, 11, 12]. These molecules have been proposed as novel non-invasive biomarkers for hepatitis C virus and hepatocellular carcinomas [13]. Additionally, there have been studies to identify microRNAs and establish their profile in bacterial infections of cattle [14, 15]. However, there has not been a study to establish microRNA profiles in cattle exposed to *M*. *bovis*; therefore, our objective was to identify microRNAs associated with a serum antibody response to *M*. *bovis* in beef cattle.

## Materials and Methods

### Animals

Sera from sixteen beef steers born during the spring, 2013, were obtained from the US Meat Animal Research Center, Clay Center, Nebraska. Animals were bled on three occasions: during the summer of 2013, while in the pasture with the dam, at weaning in the fall of the same year, and during the spring of 2014. Bleeding of animals was done according to the management protocol approved by the Institutional Animal Care and Use Committee of the Institution. Blood was obtained by jugular venipuncture using a syringe. The sample was centrifuged at 1,300 X g for 25 minutes at 4°C and serum was aspirated and frozen at -20°C. Samples were shipped to the National Animal Disease Center, Ames, Iowa.

Health records for each animal were obtained. Two animals from the negative group developed bovine respiratory disease prior to weaning and did not develop it afterwards. The condition was diagnosed in eleven animals, from the positive and negative groups, after weaning. No assessment was made of the etiology of the condition.

### ELISA

Cattle sera were tested for antibodies reactive with *M*. *bovis* using a direct ELISA, as previously reported [16], with the following modifications: 0.5 ug of antigen was used per well. Anti-bovine IgG-peroxidase conjugate (KPL, Inc.), was diluted 1:3000 in wash buffer to detect cattle IgG and color development was halted after 45 min. The *M*. *bovis* isolate M23 was used as the source of antigen [17]. Pooled sera from 32 cattle naturally or experimentally infected with *M*. *bovis* (positive pool) or 25 healthy cattle (negative pool) were used as positive and negative controls. The presence or absence of serum antibody to *M*. *bovis* was confirmed in each animal using a commercially available ELISA (Biovet, Inc.) prior to selection for inclusion in the appropriate pool. Sera included in the positive pool were 3+ or 4+ positive, on a scale of 1+ to 4+, as directed by the ELISA manufacturer. The pool itself tested as 4+ with the Biovet ELISA and had a level of IgG higher than that of the positive control serum provided with the kit. A positive result in our in-house ELISA was defined as an average absorbance at 405 nm greater than the average plus 3 standard deviations of the negative control, calculated independently for each plate analyzed.

### MicroRNA isolation

MicroRNAs were isolated from the serum samples using the miRNeasy Serum/Plasma kit (QIAGEN, Germantown, MD) using 200ul of serum sample. MicroRNAs were extracted according to the manufacturer’s direction and the samples were eluted in 14ul of RNase free water. After extraction 1 ul of each sample was run using the Small RNA chip on an Agilent 2100 Bioanalyzer (Agilent Technologies, Santa Clara, CA) to quantify the microRNAs extracted from the samples. MicroRNA concentration was determined by using a 10–40 nucleotide gate.

### Library Preparation

MicroRNA preparation extracted from each sample was used to prepare sequencing libraries. The libraries were prepared using the NEBNext Multiplex Small RNA Library Prep Set for Illumina Set 1 and 2 (New England BioLabs, Ipswich, MA). The libraries were individually index with the Illumina 1–24 indexed primers. Six microliters of each animal’s small RNA fraction was used in library preparation according to the manufacturer’s instructions. After the library preparation the libraries were cleaned up and concentrated using the QIAquick PCR purification kit (QIAGEN, Germantown, MD) from 100ul to 27.5ul. The quality and quantity of the libraries were determined by running 1 ul of each library on a DNA 1000 chip on an Agilent 2100 Bioanalyzer (Agilent Technologies, Santa Clara, CA). Five nanograms of each indexed library was then pooled and size selected within a 135–170 nucleotide range. The total volume of the pool was 246.5 ul. The pool was concentrated using the QIAquick PCR purification kit (QIAGEN, Germantown, MD) to 35ul of RNase free water. The pool was then size selected using the Pippin Prep on a 3% Agarose gel without added ethidium bromide (SAGE Sciences, Beverly, MA) with a size selection of 142–170 nucleotides according to the manufacturer’s instructions. After the gel was run the pools were concentrated using the QIAquick PCR purification kit (QIAGEN, Germantown, MD) by eluting in 32 ul of RNase free water. One microliter of the size selected library pool was run using a High sensitivity DNA chip on the Agilent 2100 Bioanalyzer (Agilent Technologies, Santa Clara, CA). The concentration was determined by using a 135–170 nucleotide gate. The final concentration of the size selected pool library was 1.5nM and the pool was stored at -20°C.

### Sequencing the Library Pool

The pooled and size selected library was sequenced using the Hi-Seq Sequencing Kit v2 50 Cycles (Illumina, San Diego, CA) in the Sequencing Core Facility at the National Animal Disease Center (NADC).

### Data Analysis

The quality of Illumina sequences was inspected using FastQCv0.11.22 program in the fastx toolkit3. The Illumina adapter was removed using fastx_clipper. Sequences of bovine microRNAs and their precursors were downloaded from miRBase (v21). Reads were mapped to known bovine microRNAs, and read counts for each microRNA were compiled and normalized using miRDeep2 [18]. Sequences have been submitted to NCBI Short Read Archive4, under BioProject accession PRJNA319677.

### Statistical Analysis

Analysis was done using the Mixed procedure of SAS (SAS Inst. Inc., Cary, NC). The model included the effects ELISA status (positive or negative), season (summer, fall, or spring), and the interaction between ELISA status and season.

Probability values shown are nominal and uncorrected for multiple testing. Sixteen animals were used to ascertain the association of microRNAs with ELISA status in the present study. Additional experimental units would be needed if significance was adjusted for multiple comparisons. Although next generation sequencing allows profiling microRNAs in each experimental unit, the cost associated with embarking on a large scale study is still a limiting factor. The present study was designed to ascertain nominal significant differences with the minimal number of samples. For this reason it was deemed relevant to present un-corrected significances in this study. Significances should be taken in consideration when interpreting results.

## Results

There were 1,374,697 sequences mapped to microRNAs in the bovine genome. Of these, 1,129,750 sequences corresponded to 27 highly abundant microRNAs with more than 10,000 copies each for all 16 animals. MicroRNAs with fewer than 10,000 copies were excluded from the study. Table 1 shows the number of copies for each microRNA used in the study.

**Table 1:** Total number of copies for each microRNA in the study.

microRNA	Number of copies
bta-miR-27a-3p	10,043
bta-miR-378	11,781
bta-miR-1246	12,289
bta-miR-27b	12,882
bta-let-7a-5p	13,950
bta-miR-26a	15,393
bta-let-7b	17,432
bta-miR-191	17,573
bta-miR-10b	21,551
bta-miR-30d	25,545
bta-miR-451	26,589
bta-miR-22-3p	27,167
bta-miR-320a	29,822
bta-miR-192	29,881
bta-miR-423-3p	30,105
bta-miR-24-3p	31,264
bta-miR-21-5p	31,587
bta-miR-25	32,500
bta-miR-128	35,232
bta-miR-99a-5p	40,154
bta-miR-181a	44,182
bta-miR-140	49,070
bta-miR-148a	64,803
bta-miR-92a	101,121
bta-miR-122	106,055
bta-miR-423-5p	141,629
bta-miR-486	150,150
Total	1,129,750

There were four microRNAs associated (*P*< 0.05) with ELISA status (Table 2). For bta-let-7b and bta-miR-24-3p, the positive group had a greater number of copies when compared to the negative group; whereas for bta-miR-92a and bta-miR-423-5p, the negative group had the greatest number of counts when compared to the positive group.

**Table 2:** MicroRNA, normalized mean for each microRNA ELISA status, standard error (SE), and their association (P-value).

	Serum antibody to *M*. *bovis*			
microRNA	Negative	Positive	SE	P-value
bta-let-7b	11,691	15,421	1,200	0.0336
bta-miR-24-3p	15,908	24,390	1,495	0.0002
bta-miR-92a	83,405	64,330	4,156	0.0023
bta-miR-423-5p	124,920	101,818	6,315	0.0133

A total of 21 microRNAs were associated with season (Table 3). Six microRNAs (bta-miR-1246, bta-miR-10b, bta-miR-423-3p, bta-miR-99a-5p, bta-miR-181a, and bta-miR-423-5p), had the fewest copy numbers (*P*< 0.01) in the spring, 2014, when compared with copy numbers produced during summer and fall, 2013. There were six microRNAs (bta-miR-27b, bta-miR-191, bta-miR-30d, bta-miR-451, bta-miR-25, and bta-miR-140), that had the greatest number of copies (*P*< 0.05) in the spring, 2014, when compared with summer and fall, 2013. Three microRNAs (bta-miR-148a, bta-miR-26a, and bta-miR-21-5p), had the greatest copy numbers (*P*< 0.02) during the summer, 2013, when compared to the fall, 2013 and spring, 2014. Bta-miR-22-3p and bta-miR-24-3p had the fewest number of copies in summer, 2013, an intermediate number of sequences in fall, 2013, and the greatest number in spring, 2014 (*P*< 0.0001). Bta-miR-320a and bta-miR-192 had the greatest number of copies during fall, 2013, while spring, 2014, had the fewest (*P*< 0.02). Bta-miR-486 had the fewest counts during fall, 2013, and the highest in spring, 2014 (*P* = 0.0347), while bta-miR-122 had the lowest in summer, 2013, and the highest in spring, 2014 (*P* = 0.0143).

**Table 3:** MicroRNA, normalized mean by season, standard error (SE), and their association (P-value).

	Season				
microRNA	Summer, 2013	Fall, 2013	Spring, 2014	SE	P-value
bta-miR-1246	12,120^a^	14,458^a^	5,052^b^	2,097	0.0078
bta-miR-10b	19,337^a^	21,934^a^	10,900^b^	1,795	0.0002
bta-miR-423-3p	25,183^a^	22,061^a^	18,296^b^	1,150	0.0006
bta-miR-99a-5p	33,817^a^	39,884^a^	21,431^b^	2,452	<0.0001
bta-miR-181a	45,650^a^	40,763^a^	15,644^b^	2,812	<0.0001
bta-miR-423-5p	132,156^a^	124,392^a^	83,559^b^	7,735	0.0001
bta-miR-27b	8,131^a^	8,211^a^	10,594^b^	635	0.0126
bta-miR-191	8,965^a^	9,130^a^	16,364^b^	1,100	<0.0001
bta-miR-30d	13,286^a^	15,684^a^	25,586^b^	1,401	<0.0001
bta-miR-451	13,621^a^	13,973^a^	23,056^b^	2,735	0.0298
bta-miR-25	20,598^a^	16,282^a^	28,104^b^	2,209	0.0019
bta-miR-140	31,099^a^	32,583^a^	43,215^b^	3,181	0.0198
bta-miR-148a	57,830^a^	45,782^b^	40,240^b^	4,032	0.0115
bta-miR-26a	17,286^a^	7,709^b^	8,971^b^	1,102	<0.0001
bta-miR-21-5p	29,242^a^	19,792^b^	18,602^b^	2,705	0.0151
bta-miR-22-3p	13,232^a^	19,023^b^	24,186^c^	1,131	<0.0001
bta-miR-24-3p	11,699^a^	18,207^b^	30,541^c^	1,831	<0.0001
bta-miR-320a	22,900^a^^,^^b^	30,932^a^	17,732^b^	2,877	0.0085
bta-miR-192	21,830^a^^,^^b^	25,455^a^	17,702^b^	1,823	0.0166
bta-miR-486	109,203^a^^,^^b^	78,821^a^	119,215^b^	11,017	0.0347
bta-miR-122	44,536^a^	72,495^a^^,^^b^	105,289^b^	14,010	0.0143

^a, b, c,^ Means without a common superscript within row are statistically different (P< 0.05).

**Fig 1:**
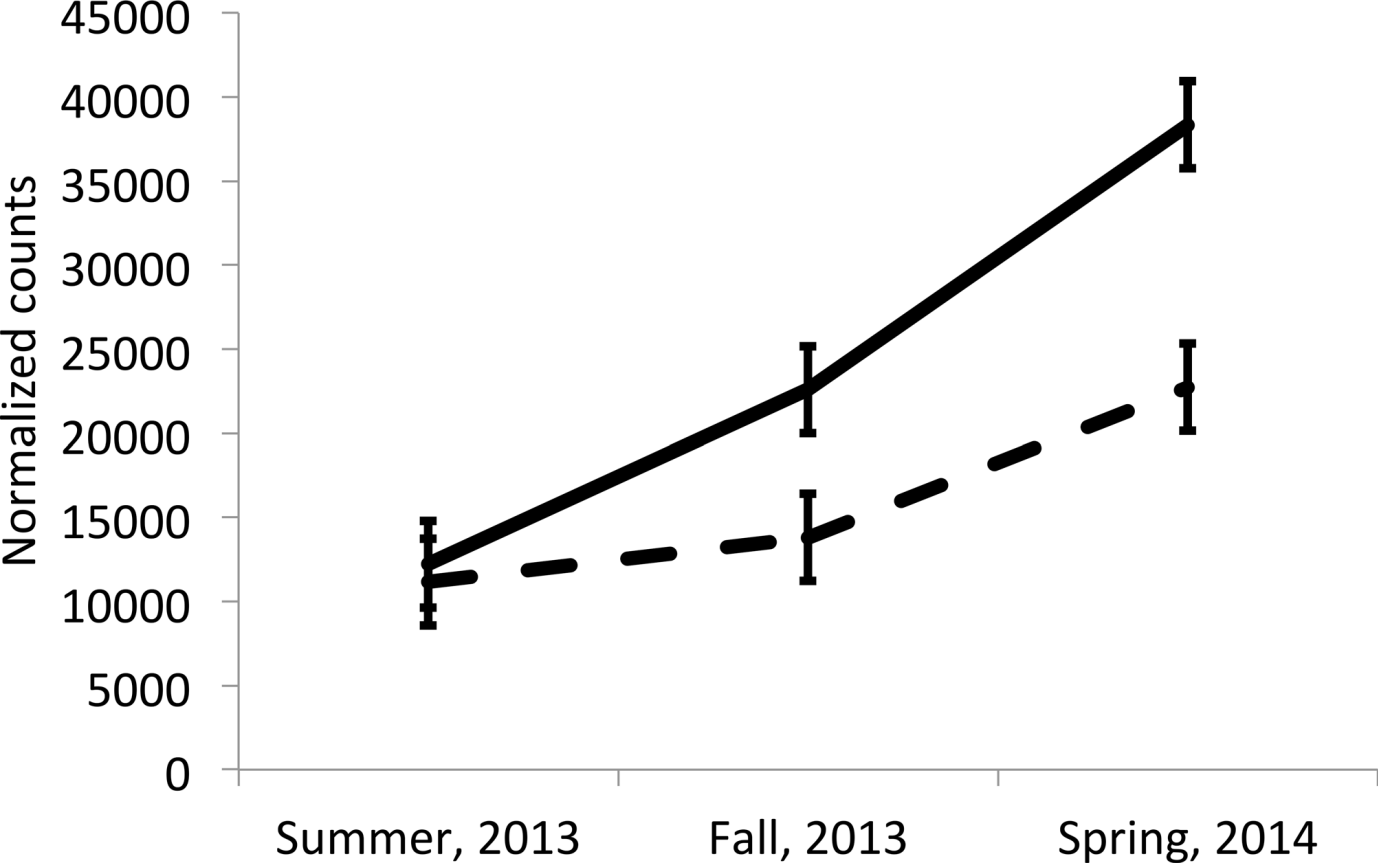
Interaction of season and antibody response to *M*. *bovis* for bta-miR-24-3p (P = 0.0268). Solid and dashed lines correspond to positive and negative groups, respectively.

Fig 1 shows the interaction (*P* = 0.0268) of ELISA status (positive and negative groups) and season for bta-miR-24-3p. During summer, 2013, both groups have similar (*P*> 0.05) copies of this microRNA. During fall, 2013, the positive group has an increased production of the microRNA, which is different from the negative group (*P* = 0.0211). In the spring of 2014, both groups increase the number of counts for this microRNA, but the differences between both groups in this season are significant (*P* = 0.0001).

Of sixteen animals in which ELISA status was determined, thirteen developed respiratory disease (two prior, and eleven post-weaning). No association between ELISA status and respiratory disease should exist. It is probable that *M*. *bovis* was involved in the development of respiratory disease in these animals, but the etiology was undetermined.

## Discussion

In this study, bta-miR-24-3p abundance increased in the seropositive group. This microRNA has been previously been reported to be associated with Heme Oxygenase-1 (HO-1) in other species. HO-1 is an enzyme that degrades heme groups in different components that give the cell anti-inflammatory, anti-oxidant and antiviral properties [19]. One of the components that HO-1 produces is carbon monoxide, which is known to be an important mediator of the host defense response mechanism [20]. For this reason, HO-1 is known as a cytoprotective enzyme that gives the cell cytoprotective properties against bacteria, viruses, and fungi [19]. Bta-miR-24-3p regulate the production of HO-1 at the post-transcriptional level [21].

Bta-miR-24-3p upregulation has been reported in challenge studies. In bovine mammary epithelial cells challenged with *E*. *coli*, an over-expression of bta-miR-24-3p was identified [22]. It has also been observed that bta-miR-24-3p was over-expressed in serum samples from patients with hepatocellular carcinoma [13]. Additionally, this micro RNA is over-expressed in swine challenged with Porcine Reproductive and Respiratory Syndrome Virus (PRRSV), and has been identified as a regulator of HO-1 [23]. It has been proposed that upregulation of bta-miR-24-3p inhibits the transcription of HO-1, therefore, hampering the cell’s ability to defends itself against pathogens [23]. Therefore, it is probable that *M*. *bovis* has a similar effect in cattle. In the present study, an upregulation of bta-miR-24-3p was detected after animals became ELISA-positive. It can be postulated that bta-miR-24-3p is over-expressed when exposed to a pathogen; however, further studies are needed to establish if HO-1 is the target of bta-miR-24-3p in *M*. *bovis* exposure in cattle.

The microRNA bta-let-7b was upregulated in the current study for the positive, compared to the negative group. A similar increase in abundance has been identified in other species with different diseases. Thornburg et al. [24], using monocyte-derived dendritic cells challenged with respiratory syncytial virus (RSV), determined that human let-7b was over-expressed in challenged versus non-challenged cells. Additionally, Edmonds and Eischen [25], indicate that let-7b was upregulated in recurrent tumors but not non-recurrent tumors, in human lungs with adenocarcinomas. Let-7b was also over-expressed in mice synaptoneurosomes during prion disease [26]. It has been reported that members of the let-7 family target, and directly inhibit, interleukin-6 (IL-6). During tumorgenesis, NF-κB mediates the inflammatory response, reducing transcription of let-7 family members. IL-6 increases when let-7 family members are depleted [27]. The upregulation of bta-let-7b in the positive group may be a mechanism of the pathogen to inhibit IL-6 production and the ability of the animal to defend itself.

Bta-miR-92a was down-regulated in the positive, compared to the negative group for the study herein. Berschneider et al. [28], using human tissue from idiopathic pulmonary fibrosis (IPF) patients, determined that this condition is mediated by the WNT1-inducible signaling pathway protein-1 (WISP1), which is a target of miR-92a. MiR-92a was down-regulated in IPF samples, while WISP1 was upregulated, and Berschneider et al. [28] provide evidence for WISP1 regulation by miR-92a in pulmonary fibrosis. Toll-like receptors (TLR) are essential for the initiation of the innate immune response, recognizing pathogens and the response of the host against them [29]. Lai et al. [30], demonstrated that stimulation of TLR production in macrophages down-regulates miR-92a. It is probable that exposure to *M*. *bovis* has a similar effect in cattle. Bta-miR-92a is a candidate to be used as a diagnostic tool in animals exposed to the pathogen.

Conflicting data have been reported on the expression of bta-423-5p in different conditions. In the present study, there was a down-regulation of the microRNA in the positive, compared to the negative group. Punga et al [31], in a study using lung samples affected with myasthenia gravis (a chronic autoimmune disorder caused by an antibody-mediated attack against neuromuscular junctions) in humans, indicate that hsa-miR-423-5p is over-expressed. Punga et al. [31], indicate that possible immunosuppression increases this microRNA. Jin et al. [22], using bovine mammary gland epithelial cells challenged with *E*. *coli*, found an upregulation of bta-miR-423-5p. Lawless et al. [32], also found this microRNA over-expressed in bovine monocytes in mammary gland affected with mastitis due to *Streptococcus uberis*. It is possible that bta-miR-423-5p may be upregulated in dairy cattle affected with mastitis, whereas circulating bta-miR-423-5p may be down-regulated in beef cattle when exposed to a pathogen that produces respiratory disease.

The ELISA used here to identify cattle as positive or negative for *M*. *bovis* is based on the reactivity of serum IgG with an extract enriched in membrane proteins of the bacterium. ELISAs of similar design are commonly used for detection of *M*. *bovis*-infected animals in both research and diagnostic settings. However, it is not known whether cross-reactive antibodies that may be elicited by infection with related commensal species, such as *M*. *bovirhinis*, could falsely contribute to estimates of *M*. *bovis*-specific antibody in individual animals, including those in this study. Nonetheless, seroconversion on a group level is predictive of *M*. *bovis* infection, especially when antibody titers are high [33], as we found here for sera from animals in the positive group. At the fall, 2013 sampling, when positive and negative groups were defined, the level of serum antibody in all 8 positive animals exceeded the level in the pool of sera used as a positive control, in most cases by at least two-fold. Therefore, we expect any cross-reactive antibodies detected by the ELISA to have had a negligible effect on the accurate identification of cattle infected with *M*. *bovis*.

The present study comprises of microRNAs that are differentially expressed throughout the growth stage of beef cattle, surveyed by season. Two studies in the Chinese Qinchuan breed evaluated microRNAs in cattle [34, 35]. A genome-wide profile was done, in which fetal and adult muscle tissues were compared to establish the genome-wide DNA methylation, microRNA and messenger RNA transcriptional activity of muscle in cattle, with the objective to understand how DNA methylation and microRNAs regulate the expression of target genes [35] had the objective to identify expression of microRNAs in bovine muscle at two prenatal, and three postnatal stages.

In the present study, bta-miR-22-3p was upregulated as the animals grew older. This microRNA was also upregulated in adult, when compared to fetal muscle tissue in the previously mentioned studies, regardless of the tissue in which it is measured [34, 35]. Bta-miR-22-3p may be a critical micro RNA associated with development of cattle.

Several microRNAs had similar expression when comparing results from the present study with those of There were nine microRNAs (bta-miR-10b, bta-miR-423-3p, bta-miR-99a-5p, bta-miR-181a, bta-miR-423-5p, bta-miR-148a, bta-miR-26a, bta-miR-192, and bta-miR-486), that were upregulated in earlier stages of life in both studies. These microRNAs may be important earlier in life, and their relevance diminishes as the animal grows older, given that similar expression patterns were identified regardless of the tissue (serum or muscle).

There were eight microRNAs (bta-miR-27b, bta-miR-191, bta-miR-30d, bta-miR-451, bta-miR-25, bta-miR-140, bta-miR-24-3p, and bta-miR-122), that were upregulated in older animals in the present study, and upregulated in fetal muscle tissue of the study. These differences in expression may be tissue-specific, and may be produced by cells and not released to circulating serum. Further studies would be needed to establish whether these differences are due to aging.

There were three microRNAs (bta-miR-1246, bta-miR-21-5p, and bta-miR-320a) that were identified in the present study, but unreported by. These microRNAs may be produced by tissues other than muscle and released to circulating serum, therefore not identified when studying muscle samples [35].

Coordinated cellular activity of different tissues in an organism is based on the ability to communicate among different cell types. MicroRNAs are molecules by which cells can communicate with each other and their impact in biological processes is becoming apparent [36, 37]. Serum is a readily available sample, and is used to monitor the health status of living organisms [13]. The use of bovine serum will allow the development of research programs focused on establishing microRNA health patterns in cattle, and to ascertain their exposure to pathogens.

There were 21 microRNAs associated with season (growth of cattle), suggesting that these microRNAs could be targets to improve productivity in the species. We also identified bta-let-7b, bta-miR- 24-3p, bta-miR- 92a, and 423-5p, to be associated (P< 0.05) with serum antibody response to *M*. *bovis* These microRNAs have been recognized as involved in the host defense mechanisms against pathogens in human, mice, and dairy cattle. Further studies are needed to establish if these microRNAs could be used as a diagnostic indicator of exposure, or whether intervention strategies could be developed to be used as an alternative to antibiotics for controlling disease due to *M*. *bovis*.
